# Training Cerebrovascular and Neuroendovascular Surgery Residents: A Systematic Literature Review and Recommendations

**DOI:** 10.31486/toj.23.0118

**Published:** 2024

**Authors:** Tyler Scullen, James Milburn, Mansour Mathkour, Angela Larrota, Oluyinka Aduloju, Aaron Dumont, John Nerva, Peter Amenta, Arthur Wang

**Affiliations:** ^1^ Department of Neurological Surgery, Tulane Medical Center, New Orleans, LA; ^2^ Department of Radiology, Ochsner Clinic Foundation, New Orleans, LA; ^3^ The University of Queensland Medical School, Ochsner Clinical School, New Orleans, LA; ^4^ International School of Louisiana, West Bank Campus, New Orleans, LA; ^5^ Soka University of America, Aliso Viejo, CA; ^6^ Department of Neurological Surgery, Medical College of Wisconsin, Milwaukee, WI; ^7^ Department of Neurological Surgery, University of Massachusetts, Worchester, MA

**Keywords:** *Education–medical–graduate*, *endovascular procedures*, *fellowships and scholarships*, *internship and residency*, *neurosurgical procedures*, *specialization*

## Abstract

**Background:** The rapid evolution of neuroendovascular intervention has resulted in the inclusion of endovascular techniques as a core competency in neurosurgical residency training.

**Methods:** We conducted a literature review of studies involving the training of neurosurgical residents in cerebrovascular and endovascular neurosurgery. We reviewed the evolution of cerebrovascular neurosurgery and the effects of these changes on residency, and we propose interventions to supplement contemporary training.

**Results:** A total of 48 studies were included for full review. Studies evaluated trainee education and competency (29.2%, 14/48), neuroendovascular training models (20.8%, 10/48), and open cerebrovascular training models (52.1%, 25/48), with some overlap. We used a qualitative analysis of reviewed reports to generate a series of suggested training supplements to optimize cerebrovascular education.

**Conclusion:** Cerebrovascular neurosurgery is at a crossroads where trainees must develop disparate skill sets with inverse trends in volume. Continued longitudinal exposure to both endovascular and open cerebrovascular surgical fields should be mandated in general resident education, and blended learning tactics using adjunct simulation systems and models should be incorporated with didactics to both optimize learning and alleviate restraints placed by decreased volume and autonomy.

## INTRODUCTION

The rapid evolution of neuroendovascular intervention has resulted in a significant shift in the management of cerebrovascular pathology.^1,2^ Accordingly, subspecialization and the inclusion of endovascular techniques as a core competency have been introduced into neurosurgical residency training.^3-9^ Young cerebrovascular surgeons face a transition in the field that is populated by a heterogeneous group: those who are dual-trained in open and endovascular techniques and those who subspecialized solely in one or the other.

In this literature review, we investigate the evolution of cerebrovascular neurosurgery during the era of endovascular intervention and the effects of these changes on residency training. We propose intensive training interventions using blended learning strategies and pedagogic principles of the universal design for learning (UDL) framework to supplement contemporary residency training. The UDL, an increasingly common pedagogy used across multiple educational settings, aims to teach multiple trainees with unique learning styles and experiences within a single curriculum.^10^ UDL strategies incorporate multimodal approaches in trainee engagement, information presentation, and trainee expression of learning in a blended format to allow students to thrive regardless of background or learning style.^10^

## METHODS

We conducted a systematic literature review without meta-analyses, querying the United States National Library of Medicine at the National Institutes of Health PubMed database and common internet search engines for studies involving the training of neurosurgical residents in cerebrovascular and endovascular neurosurgery. We found 134 results using the medical subject heading (MeSH) keywords “endovascular,” “cerebrovascular,” and “neurosurgery residency” on November 7, 2022. Studies were included if they presented data regarding assessments of cerebrovascular or endovascular education or simulation techniques in the context of neurosurgical resident or fellowship training. Studies were excluded if (1) education consisted entirely of fellows or residents in programs other than neurological surgery; (2) they were opinion papers or literature reviews without original data; (3) data were presented regarding the feasibility of a particular technique or simulation prior to its application in active education; or (4) they presented data regarding subspecialties outside of neuroendovascular or cerebrovascular surgery. Forty-eight studies met our inclusion criteria and were selected for full review (Figure). This study was not registered, and a protocol was not prepared.

**Figure.**
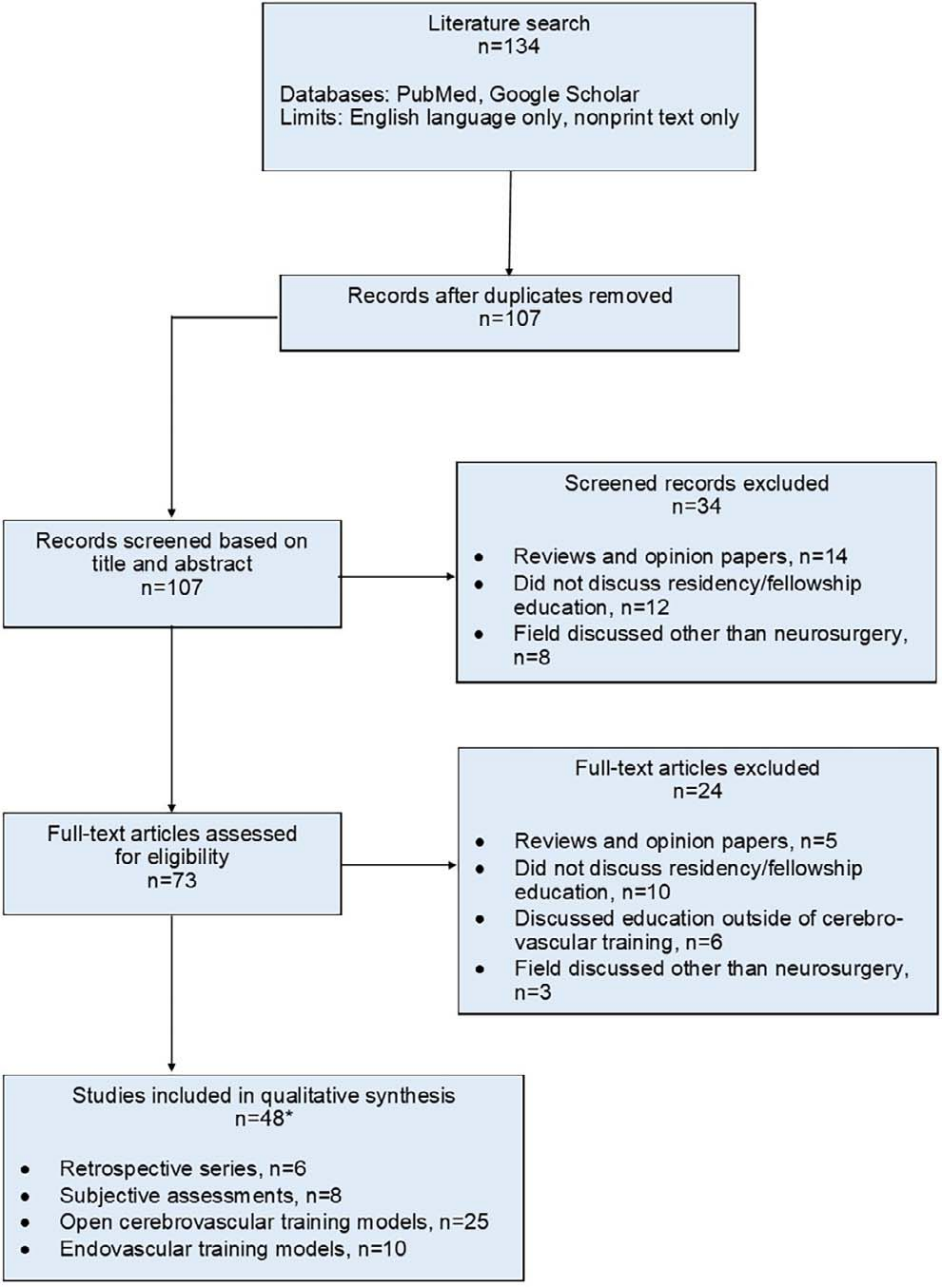
**Flowchart of literature search methodology.** *One study is included in two categories.

## STUDIES ASSESSING COMPETENCY AND TRAINING TECHNIQUES

We sorted the 48 studies into 3 categories assessing resident and fellow competency and training techniques.

The first category of studies (29.2%, 14/48) includes studies evaluating trainee education and competency (Table 1).^4,5,11-22^ These studies queried the perceived ability, experience, and clinical knowledge of participants relevant to cerebrovascular disease through either subjective assessments or questionnaires (57.1%, 8/14),^4,5,17-22^ review of case logs (21.4%, 3/14),^14-16^ or direct evaluation in cases or using models (21.4%, 3/14).^11-13^ Reports reviewing case logs unanimously described decreases in open cerebrovascular volume and increases in endovascular volume during residency,^14-16^ with the endovascular field seeing increased enrollment in postgraduate fellowship.^4,5^ All reports assessing alternative practical training methods outside of the operating room reported positive results with a range of models, simulations, didactics, and direct feedback.^11-13,17-22^

**Table 1.** Neurosurgical Resident Assessments

Study	Tool	Type	Assessment Subject	Major Findings
Aoun et al, 2015^11^	Northwestern Objective Microanastomosis Assessment Tool	Retrospective review	Construct validity	Face and construct validity established
Belykh et al, 2017^12^	Aneurysm clipping model			Face and construct validity established
Shin et al, 2013^13^	Resident transfemoral catheterization		Procedural complications	No complications observed across 465 selective angiograms
Fredrickson et al, 2019^14^	Resident open cerebrovascular case number review		Training exposure	Median 80 open aneurysm cases in CAST programs
Bowden et al, 2021^15^	Resident case log entry			Significant decreases in open cerebrovascular cases noted with time
Agarwal et al, 2018^16^	Resident case log entry			Increased endovascular cases and decreased open cases reported with time
Strozyk et al, 2010^17^	Program director fellow and resident assessment	Subjective assessment		Increased neurosurgery and neurology and decreased radiology residents pursuing endovascular fellowship
Lanzino and Rabinstein, 2011^5^	Endovascular surgeon questionnaire			Variable practice patterns recorded for given pathologies across endovascular surgeons
Chalouhi et al, 2015^4^	Neuroendovascular fellowship survey			Only 42% of surveyed fellows able to get femoral access at time of starting fellowship
Rubino et al, 2014^18^	2D vs 3D anatomy imaging survey		Anatomic education	Discordance observed in respondents’ ability to recognize 2D vs 3D anatomy
Zammar et al, 2015^19^	Pre- and post-course resident survey		Clinical knowledge	Residents favored the viability of simulation in training
Koizumi et al, 2021^20^	Multisource video recording resident assessment		Procedural knowledge	Residents reported utility in simultaneous recording of operator's hands and fluoroscopic images for procedural learning
Shakir et al, 2018^21^	Fellow and resident self-assessment		Fellowship utility	Respondents reported the greatest benefits of the fellowship to be increased job competitiveness and improved endovascular skills
Oliveira et al, 2019^22^	Microsurgical intraoperative video error review		Operative ability	Fine microsurgical dissection of the arachnoid membrane and aneurysm sac were the most improved tasks among the 7 common operative mistakes

2D, 2-dimensional; 3D, 3-dimensional; CAST, Committee on Advanced Subspecialty Training.

The second category of studies (20.8%, 10/48) includes studies describing neuroendovascular training models (Table 2).^6,8,23-30^ The studies described experiences using commercially available simulation systems (60.0%, 6/10),^6,8,23-26^ synthetic constructs (30.0%, 3/10),^27-29^ and a live animal model (10%, 1/10)^30^ in technical training and case preparation for diagnostic and interventional procedures. Simulations included the use of live fluoroscopy in 40% (4/10) of reports,^27-30^ with the remainder stressing radiation safety and exposure purely via virtual fluoroscopy times.^6,8,23-26^ All included studies involving simulations reported improvements in pretest and posttest score assessments of given simulated tasks, and comparisons of the described systems to real-life scenarios were positive.^6,8,23-26^ All studies were heavily limited in either being restricted to a specific system or model or by relying on subjective comparison.

**Table 2.** Neuroendovascular Training Models

Field	Study	Type	Model	Fluoroscopy	Skill Set
Angiography	Fargen et al, 2012^8^	Simulator	VIST-C Simulator Systems^a^	Simulated	Diagnostic angiography
	Spiotta et al, 2013^6^		Simbionix simulator^b^		Diagnostic angiography
	Zaika et al, 2016^23^		Simbionix simulator^b^		Diagnostic angiography
	Pannell et al, 2016^24^		Simbionix simulator^b^		Interventional angiography
	Dardick et al, 2019^25^		Simbionix plus VR		Interventional angiography
	Patchana et al, 2020^26^		VIST G5^c^		Interventional angiography
	Sugiu et al, 2004^27^	Synthetic	Silicone	Live	Interventional angiography
	Miranpuri et al, 2014^28^		Silicone		Diagnostic angiography
Flow diversion	Sullivan et al, 2018^29^		3D-printed replicator		Pediatric fusiform aneurysm treatment
Recanalization	Lv et al, 2020^30^	Animal	Anesthetized canine		Mechanical thrombectomy

^a^VIST-C Simulator Systems (Mentice).

^b^Simbionix simulator (Simbionix USA Corp).

^c^VIST G5 simulator (Mentice).

3D, 3-dimensional; VR, virtual reality.

The third category of studies (52.1%, 25/48) includes studies describing open cerebrovascular training models (Table 3; Belykh et al^12^ is included in both the first and third categories).^12,31-54^ This largest group of reviewed reports included a wide variety of synthetic and living models using human and animal tissues, as well as various combinations, to best create lifelike simulations of microsurgical cerebrovascular conditions. Two studies (8.0%)^31,32^ discussed training models in the context of vascular injury, 20 studies (80.0%) discussed aneurysm surgery,^12,33-51^ and 4 (16.0%) discussed microvascular anastomoses^51-54^ (de Oliveira et al^51^ addressed both aneurysm surgery and microvascular anastomoses). Models used various technology resources and ranged from simple 3-dimensional (3D)-printed aneurysm replicas to complex patient-specific models implanted into perfused human cadavers. All studies reported intuitive improvements in tasks completed using anatomic training models, and although participating residents gave favorable responses, experienced surgeons who were queried reported considerable mechanical differences compared to live procedures.^12,31-54^ The queried surgeons also cautioned that the best use of anatomic models would likely be as a surrogate rather than a replacement of live operative education.^12,31-54^

**Table 3.** Open Cerebrovascular Training Models

Field	Study	Type	Model	Skill Set
Trauma	Ciporen et al, 2018^31^	Human tissue	Perfused cadavers	Vascular injury
Trauma	Zada et al, 2018^32^	Human tissue	Perfused cadavers	Vascular injury
Aneurysm surgery	Aboud et al, 2015^33^	Human tissue	Perfused cadavers	Aneurysm rupture
Aneurysm surgery	Scholz et al, 2008^34^	Animal tissue	Rat arterial vessels	Aneurysm clipping
Aneurysm surgery	Benet et al, 2015^35^	Hybrid	3D-printed replicator in perfused human cadavers	Aneurysm clipping
Aneurysm surgery	Alaraj et al, 2015^36^	Simulator	Virtual reality with haptics	Aneurysm clipping
Aneurysm surgery	Agarwal et al, 2012^37^	Simulator	Virtual reality with haptics	Aneurysm clipping
Aneurysm surgery	Gmeiner et al, 2018^38^	Simulator	Virtual reality with haptics	Aneurysm clipping
Aneurysm surgery	Hendricks et al, 2018^39^	Simulator	3D-printed replicator and virtual reality with haptics	Aneurysm clipping
Aneurysm surgery	Bairamian et al, 2019^40^	Simulator and synthetic	3D-printed replicator	Aneurysm clipping
Aneurysm surgery	Wurm et al, 2011^41^	Synthetic	3D-printed replicator	Aneurysm clipping
Aneurysm surgery	Mashiko et al, 2015^42^	Synthetic	3D-printed replicator	Aneurysm clipping
Aneurysm surgery	Joseph et al, 2020^43^	Synthetic	3D-printed replicator	Aneurysm clipping
Aneurysm surgery	Eftekhar et al, 2005^44^	Synthetic	Play dough	Aneurysm clipping
Aneurysm surgery	Oliveira et al, 2014^45^	Human tissue	Placental vessels	Aneurysm clipping
Aneurysm surgery	Belykh et al, 2017^12^	Human tissue	Placental vessels	Aneurysm clipping
Aneurysm surgery	Lan et al, 2016^46^	Synthetic	3D-printed replicator	Aneurysm clipping
Aneurysm surgery	Wang et al, 2018^47^	Synthetic	3D-printed replicator	Aneurysm clipping
Aneurysm surgery	Wang et al, 2018^48^	Synthetic	3D-printed replicator	Aneurysm clipping
Aneurysm surgery	Mashiko et al, 2017^49^	Synthetic	3D-printed replicator	Aneurysm clipping
Aneurysm surgery	Nagassa et al, 2019^50^	Synthetic	3D-printed replicator	Aneurysm clipping
Aneurysm and bypass surgery	de Oliveira et al, 2018^51^	Human tissue	Placental vessels	Aneurysm clipping and microvascular anastomosis
Bypass surgery	Oliveira et al, 2018^52^	Human tissue	Placental vessels	Microvascular anastomosis
Bypass surgery	Mullarkey et al, 2022^53^	Animal tissue	Chicken brachial, radial, and ulnar arteries	Microvascular anastomosis
Bypass surgery	Abla et al, 2011^54^	Animal tissue and synthetic	Chicken and turkey brachial arteries, silastic tube model	Microvascular anastomosis

## CURRENT ASSESSMENT OF CEREBROVASCULAR TRAINING

Cerebrovascular neurosurgery is increasingly managed via minimally invasive endovascular procedures,^55-58^ so current trainees must obtain competency in 2 separate technical skills prior to practice.^56^ Despite annual increases in neuroendovascular volume at teaching facilities, exposure is often limited to condensed rotations and/or fellowship training.^57,59-63^ Conversely, teaching opportunities in open techniques have steadily declined annually to the point where program minimum case requirements have shifted to prevent centers from losing accreditation.^9,56,57^ As such, many programs have trialed various supplemental educational tools, ranging from complex simulators to anatomic models.

Multiple studies have investigated trainee proficiency in the endovascular and microsurgical fields through surveys, case logs, and subjective assessments.^4,5,11-22^ Case log assessments highlighted the inverse relationship of rising endovascular and decreasing open volumes.^14-16^ Multiple reports suggested that the decrease in open cases was adequately supplemented with simulated training models.^11,12,18-20,22^ However, open cerebrovascular surgery is technically complex, requiring significant repetition and frequent exposure.^64-68^ Complex cases such as basilar apex aneurysms or high-flow anastomoses may be exceedingly rare in many programs.^69,70^ Insufficient education in such methods may result in biases to recommend suboptimal treatments because of a lack of comfort in performing complex open techniques.^69,70^ Although declining, cases requiring complex microsurgical treatment are not extinct, mandating technical proficiency to provide the best patient care.^69,70^ Despite a potential need for increased open cerebrovascular fellowship training, the number of active open fellowship programs remains limited to 12 centers, 2 of which were reported to be on probation at the time this report was written.^71^ Given decreased open case volumes, many reports describe positive resident feedback and technical improvements following completion of didactics and skilled tasks in a laboratory setting.^11,12,15,19,22^

Neuroendovascular fellowship training has seen a rise in fellowship enrollment and case volume.^4,5,14,17,21^ Reviewed assessments indicate that fellowship training was most often pursued because of a lack of exposure during residency.^4^ Respondents reported a lack of proficiency in basic angiographic skills, including arterial access and closure, vessel selection, and the ability to perform diagnostic angiograms,^4^ all of which are now considered core competencies for general neurosurgical residency.^3,4,9^ However, although 91% of surveyed programs reported resident exposure to endovascular techniques, only 26% of endovascular neurosurgeons achieved core competency during residency.^17^ In one study, no patient complications were reported for 112 diagnostic procedure cases conducted by a single resident.^13^ Although generalization of this inherently biased report is inappropriate, and complications should be statistically expected during angiography as with any procedure, this result suggests that basic and safe endovascular skill set acquisition is achievable in residency.

Although fellowship training provides invaluable experience, using fellowship training to compensate for inadequate exposure during residency may prove detrimental. Neurosurgical residency is 7 years, the maximum number allotted by the Accreditation Council for Graduate Medical Education.^72^ Fellowships delay compensation, board certification, and practice development.^61,62^

Enfolded fellowships—subspecialized training completed prior to graduation from residency—historically occur during the first half of residency training and may therefore place a significant gap between the isolated period of training and the beginning of practice.^17^ Accordingly, leadership committees have set standards requiring many enfolded fellowships to occur during the final year of residency, functionally condensing general neurosurgical education to 6 years and subspecialty training to 1 year.^64^

Segmentation of skill set education into intensive, singular blocks without repetition harms continuous learning and fails to promote generalization of information.^65-68^ Trainees may begin to become proficient at a given task during one rotation before suddenly moving on to a very different task during the next rotation.^59,62,65-68^ This piecemeal method of learning, while allowing for focused efforts in understanding complex subjects, alienates context and application to broader concepts and strategies.^10^ One strategy involves considering the angiography suite no different than the operating room, providing longitudinal exposure. Additionally, introducing supplementary tools via simulators, models, or live feedback may be beneficial to increase learning efficacy when case exposure opportunities may be relatively rare.^11,12,18-22^ However, the rarity of resident exposure to endovascular procedures is not a consequence of case volume, which is more than sufficient across all programs to achieve core competencies.^71,72^ Pathologies that can be treated by endovascular methods continue to rise annually and in proportion to neurovascular subspecialization.^3-5^

### Endovascular Simulation

Complex endovascular simulation platforms allow trainees to rehearse technical skills and operative decision-making in a no-risk environment.^6^ When combined with supervision and mentorship, these simulation experiences appear to provide an efficient training adjunct.^6^

Computer-based simulations provide a range of clinical scenarios and approximations of how various catheters and wires will behave.^7^ Trainees can select anatomic and pathologic conditions and choose between simulated catheters, interventional devices, and stents in rehearsed interventions.^6-8,23-26^ Simulation platforms can emulate varying physical properties and provide haptic and visual feedback.^6-8^ These effects are reportedly accentuated when used synergistically with alternate platforms such as virtual reality (VR).^25^ The impact on real-life preparedness and the transferability of simulated to live angiography are not well reported however, and improved scores in simulated angiography alone do not imply clinical proficiency.

Synthetic models mimicking vascular anatomy can be used under live fluoroscopy with real catheters in an angiography suite.^27-29^ Anesthetized animals, although more complex logistically, also provide realistic fluoroscopic models that are relatively affordable compared to simulation and VR systems.^30,73^ Use of live fluoroscopy mandates standardized education in radiation safety, medically critical for anyone seeking to obtain neuroendovascular proficiency, but such training may otherwise be underemphasized in neurosurgical and neurological education.^27,28,74^

### Microsurgical Simulation

Decreased open case volumes risk decreased familiarity with operative experience and planning. Illustratively, a true appreciation for the relationship between an aneurysm and its parent vessel, the selection of the correct clip configuration, and the location and projection of the dome are all variables that require experience and repetition to gain mastery.^51,52,57^

In a method conceptually similar to that of endovascular platforms, microsurgical anatomy can be reconstructed using 3D printing methods to model vascular anatomy obtained via imaging.^40,41,46^ These constructs allow the trainee to rotate the vascular tree and observe the pathology from multiple vantage points, and when combined with surrounding printed extracranial and cranial tissues, allow rehearsal of skills such as clip placement, craniotomy selection, and corridor development.^35,40-43^ Sophisticated models such as perfused human cadavers and hybridization of cadavers and synthetic material have also been advocated as useful adjuncts in combination with didactics and clinical experience.^31-33^

VR programs emphasize microsurgical 3D relationships^36-40^ and provide haptic feedback and visual immersion.^36-40^ Trainee VR surgical trajectories were reported to correspond with the majority of intraoperative trajectories, and in a small study in Austria, 94% of trainees believed simulators should be included in residency training.^38,39^ Reports also reasonably conclude that VR is not an acceptable facsimile of cerebrovascular surgery^38,39^ and note that brain manipulation, arachnoid dissection, and cerebrospinal fluid management are not accurately represented in many systems.^36-40^

In contemporary training, traditional animal models have been largely supplanted by living tissue models to mimic the challenges of microsurgery.^21,31-34,45^ Human placental vasculature is similar in size to cerebral vessels and can be easily dilated or reconstructed with balloon or open angioplasty to simulate aneurysms, fistulae, and other pathologies.^51,52^ Placental tissue in particular mimics the haptics of arachnoid and parenchymal planes.^51,52^ Models using avian and murine vessels have also received positive reports for training residents for technical skills necessary for extracranial-intracranial anastomosis and bypass.^53,54^

## RECOMMENDATIONS FOR CONTEMPORARY CEREBROVASCULAR EDUCATION

We used data from the reviewed reports (Tables 1 to 3) to generate a blended learning strategy using the UDL framework^10^ in a manner relevant to contemporary cerebrovascular education (Table 4). We organized factors incorporating multidisciplinary critical thinking, dual procedural exposure, and the use of training models and simulations in a UDL style^10^ to provide recommendations to access, build, and internalize necessary skill sets while considering trainee engagement, information representation, and expression of learning and development. The framework is intended to be applicable to cerebrovascular education in both general neurosurgical and specialty training at the residency and/or fellowship level.

**Table 4.** Recommended Intensive Training Guidelines to Assist Dual Cerebrovascular Education

Aspect	Engagement	Information Representation	Expression of Learning and Development
Access	Case logs routinely monitored by faculty Continuous cerebrovascular exposure in residency Continuous endovascular exposure in residency Elective rotations with intensive open and endovascular patient and case experience provided Trainees with deficient cerebrovascular volume assigned to needed cases	Trainees participate in endovascular (on multidisciplinary services) and open cases and learning opportunities throughout residency in the same format as other specialties in the field Trainees are educated in angiography suite layout, catheter system basics, biplanar fluoroscopy operation, basic radiation safety, and endovascular biomechanics during allocated didactic sessions	Residents who are unable to routinely participate in appropriate numbers of endovascular or open procedures allocated to dedicated adjunct learning Trainees with low case volumes or extended absence in either open or endovascular cases assigned to needed level-appropriate cases until corrected Resident case assignments may become disproportionate across specialties because of imbalances and exposure, so potential interest among senior and junior residents requires regular review with service chiefs
Build	Regular feedback on technical mastery in cerebrovascular cases Regular feedback on technical mastery in endovascular cases Feedback provided by non-neurosurgical interventionalists and neurosurgeons in relevant settings	Access to surgical and angiographic videos, as well as book funds for print and online resources provided Complications in care and technical complications illustrated through didactic sessions, morbidity and mortality, and operative videos in a constructive manner Treatment choices reviewed with individual trainees	Trainees provided with access to adjunct training systems in addition to live case participation, including Endovascular simulations Open simulations Interactive anatomic/flow models Cranial cadaver lab access
Internalize	Motivation optimized through scheduled performance reviews Self-assessment and reflection promoted in complex cases and in treatment choices in endovascular and open scenarios	Trainees regularly evaluated on ability to understand 3D cranial anatomy using angiographic images and vice versa to promote information transfer and generalization and background knowledge recall Trainees evaluated on approach constraints and biomechanical consequences of various treatments for a given pathology	Engagement of caring for cerebrovascular disease as a whole, in addition to technical skills, promoted Proposed individual case treatment strategies reviewed with trainees, discussing implications of technical abilities, treatment approaches, clinical scenario, and consequences of actions and strategies

3D, 3-dimensional.

To adequately train dual endovascular and microsurgical cerebrovascular surgeons capable of providing situationally optimal treatment, as well as to ensure cerebrovascular competency during general neurosurgical residency, we recommend additional training venues outside of live experience via validated models and simulations.^6,8,12,23-45,47-50^ We suggest using a UDL-style framework when incorporating teaching tools, as blended methods have demonstrated increased educational efficacy in academic pedagogy.^75-78^

Throughout residency, efforts should be made to longitudinally involve resident participation in level-appropriate cases conducted by both neurosurgeons and their interventional colleagues to ensure adequate and heterogeneous teaching opportunities. As with any other surgical training program, multidisciplinary didactics on endovascular tenets and radiation safety should be considered a regular part of the academic curriculum, considering the historic deficits in these areas.^4,9,13,17^

While time constraints exist in any program, neurosurgery residency is 7 years. In scenarios where resident case exposure is deficient, resident time allocation should be restructured, potentially removing the need for general neurosurgical residents to undergo fellowship training to meet core competency. For example, advanced practice providers or hospital employees can easily manage several time-consuming service tasks if performing these tasks is at the expense of residents’ practical experience.^79^

Concerning endovascular intervention, in centers where much of the case volume is handled by interventional neuroradiologists, neurologists, or cardiologists, interdisciplinary cooperation becomes necessary.^4,80^ Inherently, practitioners in individual fields exhibit a degree of competitiveness, with scattered reports from all sides discussing various specialties trying to gain ground on others.^80,81^ While competition is certainly a driving force for health care advancement, benefit is gained from collaborative multidisciplinary teamwork.^82^

Multidisciplinary cooperation is particularly relevant to endovascular fellowship programs, where trainees may have prior training in nonsurgical specialties such as interventional cardiology, vascular neurology, and interventional radiology.^17,83^ The marked differences among fellows in primary postdoctoral training is a further indication for UDL-structured frameworks that attempt to allow for equal learning curves among classmates with variable academic backgrounds and training styles.^10^

Critically, nonsurgical fellows must be diligently educated in recognizing and avoiding bias. Just as the intuition of a neurosurgeon trained only in open methods may be to operate on a given case despite better-suited endovascular options, so too nonsurgical interventionalists may be tempted to offer endovascular treatment in cases that may be better managed with open surgery. Continual emphasis must be placed on providing the best patient care in cerebrovascular disease.

Programs with large resident complements can subsegment residency training to allow residents to rotate for several contiguous months on a given specialty.^84-86^ This structure allows a trainee to work closely with one or more staff of the same specialty.^84-86^ The focused repetition within the constant discipline provided by the same trainers allows trainees to cement new experiences, achieve technical competency, increase trust with educators, and thereby increase operative autonomy.^84-86^ Importantly, these rotations should be intermittently repeated throughout training, allowing interval longitudinal development that periodically reinforces learned skills.^84-86^

In the context of cerebrovascular training, neurosurgery residents may benefit from periodic rotations during which they participate in open cases with neurosurgery staff and in endovascular cases with multidisciplinary services to promote real-world conditions. Alternatively, the concept of developing specialized tracks in residency, in which residents can pursue multiyear continuous training in their desired specialty is under active investigation, but no analysis of outcomes or impacts on general education and academic development has yet been done.^62^

## LIMITATIONS

We conducted a literature review on current strategies and assessments in contemporary cerebrovascular surgery training. Many of the included reports are inherently biased given their structure as subjective assessments or surveys or evaluation of various training techniques using pre- and post-training scores. Data and methods on how to objectively qualify the degree to which adjunct training platforms can be clinically translated to live operative skills are limited. The recommendations offered throughout this discussion are the educated opinions of the authors based on reviewed reports. Continued academic focus on cerebrovascular and neurosurgical pedagogy in an objective manner is highly warranted.

## CONCLUSION

Cerebrovascular neurosurgery is at a crossroads in which trainees wishing to obtain competency must develop disparate skill sets with inverse trends in case availability. Continued longitudinal exposure to both endovascular and open cerebrovascular fields should be mandated in general resident education. Blended UDL-style learning tactics using multiple training modalities should be considered to both optimize learning and alleviate restraints placed by decreased volume and autonomy. The primary author's opinion is that the inability of a graduating neurosurgery resident to perform basic endovascular and microsurgical cerebrovascular procedures is a failure of core competency education. Fellowship training should serve to fine-tune previously developed skills, not to buttress insufficient experience.

## Notes

*This article meets the Accreditation Council for Graduate Medical Education and the American Board of Medical Specialties Maintenance of Certification competencies for Patient Care, Medical Knowledge, and Practice-Based Learning and Improvement.*
